# Human metaphase chromosome consists of randomly arranged chromatin fibres with up to 30-nm diameter

**DOI:** 10.1038/s41598-020-65842-z

**Published:** 2020-06-02

**Authors:** Toshiyuki Wako, Akiyo Yoshida, Jun Kato, Yuji Otsuka, Shinichi Ogawa, Kohei Kaneyoshi, Hideaki Takata, Kiichi Fukui

**Affiliations:** 1 Institute of Crop Sciences, National Agriculture and Food Research Organization, 2-1-1 Kannondai, Tsukuba, Ibaraki, 305-8602 Japan; 2 Morphological Research Laboratory, Toray Research Center Inc., 3-3-7 Sonoyama, Otsu, Shiga, 520-8567 Japan; 3 Nanoelectronics Research Institute, National Institute of Advanced Industrial Science and Technology, 1-1-1 Umezono, Tsukuba, Ibaraki, 305-8568 Japan; 4 Graduate School of Engineering, Osaka University, 2-1 Yamadaoka, Suita, Osaka, 565-0871 Japan; 5 Kansai Center, National Institute of Advanced Industrial Science and Technology, Midorigaoka, Ikeda, Osaka, 563-8577 Japan; 6 Graduate School of Pharmaceutical Sciences, Osaka University, 1-6 Yamadaoka, Suita, Osaka, 565-0871 Japan

**Keywords:** Chromosomes, Cytogenetics, Scanning electron microscopy

## Abstract

During cell division, mitotic chromosomes assemble and are equally distributed into two new daughter cells. The chromosome organisation of the two chromatids is essential for even distribution of genetic materials. Although the 11-nm fibre or nucleosome structure is well-understood as a fundamental fibrous structure of chromosomes, the reports on organisation of 30-nm basic chromatin fibres have been controversial, with debates on the contribution of 30-nm or thicker fibres to the higher order inner structure of chromosomes. Here, we used focused ion beam/scanning electron microscopy (FIB/SEM) to show that both 11-nm and 30-nm fibres are present in the human metaphase chromosome, although the higher-order periodical structure could not be detected under the conditions employed. We directly dissected the chromosome every 10-nm and observed 224 cross-section SEM images. We demonstrated that the chromosome consisted of chromatin fibres of an average diameter of 16.9-nm. The majority of the chromatin fibres had diameters between 5 and 25-nm, while those with 30-nm were in the minority. The reduced packaging ratio of the chromatin fibres was detected at axial regions of each chromatid. Our results provide a strong basis for further discussions on the chromosome higher-order structure.

## Introduction

Mitotic metaphase chromosomes with two chromatids are composed of two chromatin fibres, each of which is a complex of one DNA double strand and histone proteins^1^ making up the nucleosome, the fundamental structure of the 11-nm nucleosome fibre^2^. The nucleosome has a cylindrical shape of 6-nm height and 11-nm diameter on a DNA fibre, which is referred to as a beads-on-a-string structure. This structure is employed to make up the higher-order chromatin fibre with a 30-nm diameter. Because 30-nm fibrous structures have been repeatedly observed under scanning electron microscopy (SEM) at the surface of metaphase chromosomes^3^, they have been referred to as the basic chromatin fibre. Several attempts have been made to explain the 30-nm structure using various folding patterns of the 11-nm nucleosome fibre, with solenoid^4^ and zig-zag^5^ being the two representative models. However, results using cryo-electron microscopy (cryo-EM) invalidated the presence of not only the 30-nm fibre but also of any higher-order structure in the chromosome^6^, despite several models with their own experimental data being proposed^7–11^. Using ChromEM tomography, 5–24-nm chromatin fibres were recently detected^12^. To clarify these inconsistencies and to elucidate the presence of higher-order fibrous structure of chromatin, including the 30-nm fibre, which would be essential for the modelling of the chromosome structure, we used focused ion beam/scanning electron microscopy (FIB/SEM). Sequential milling by focused ion beam with nanometre steps and image acquisition supply serial section images of chromosome with high resolution^13–15^. Furthermore, application of FIB/SEM allowed us direct visualisation of the inner structure of the chromosome.

## Results

**Figure 1:**
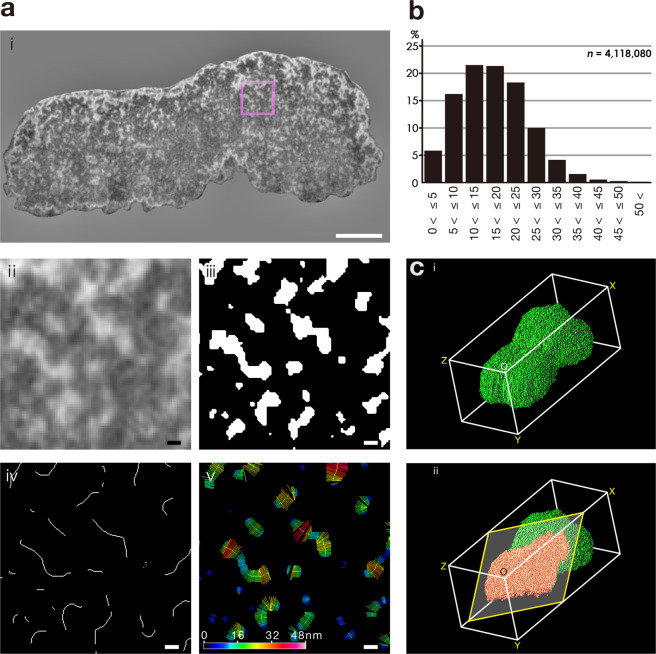
3D reconstruction of a chromosome surface and inner structure. (**a**) (i–v), Imaging processes for measuring the diameter of chromatin fibres. Bar = 500 nm (i), 25 nm (ii–v). (**b**), Histogram showing distribution of diameters of chromatins (*n* = 4,118,080). (**c**), **(i** and **ii)**, 3D reconstruction of chromosome images drawn by Volume Viewer 2.01 plugin of ImageJ 1.51 and Adobe Photoshop CC for surface and inner structure observation, respectively.

We directly dissected and observed by FIB/SEM a total of 224 cross-section images of a human metaphase chromosome coated with platinum on an aluminium substrate with 10-nm intervals (Supplementary Fig. 1). Chromosomes were isolated under conditions that minimized structural disruption using the polyamine method and ionic liquid method^14,15^. Both the chromosome index and chromosome length measured (Suppl. Fig. 1) were the closest to those of human chromosome 20^16^ that contains 64.4-Mbp of DNA^17^ per chromatid. Figures 1a-i and ii show a whole cross-section image and its partially enlarged cross-section image, respectively. A total of 164,105 chromatin fibres were extracted as white regions in binary images (Fig. 1a-iii). The chromatin fibres occupied 19% of the chromosome volume, and DNA density was estimated to be 16-Mbp/µm^3^. The chromatin fibres showed various thickness, length, branching, and bending. Axial lines for individual chromatin regions were detected by applying a line thinning procedure throughout the chromosome (Fig. 1a-iv). A total of 4,118,080 lines orthogonal to axial lines at 152,556 chromatin fibres were generated, which were variously pseudo-coloured based on their lengths (Fig. 1a-v). When the length was approximately 12, 24, and 36-nm, the line was coloured cyan, yellow, and red, respectively. Figure 1b presents the distribution frequencies of the lengths of measured chromatin diameters obtained by the above method. First, the number of chromatin fibres with diameter >25-nm were the minority, indicating that thick chromatin fibres were not involved in the construction of higher-order structures of chromosomes at the metaphase stage. Second, the mean value of diameters of the chromatin fibres was 16.9 ± 8.2 nm, suggesting the presence of both the 11-nm and 30-nm chromatin fibres (Fig. 1b). The diameter of chromatin fibres within the 4 major classes ranging from over 5 to 25-nm occupied 77.8% in total. Random arrangement of nucleosomes would constitute chromatin fibres in the construct of the metaphase chromosome. To obtain the authentic chromosome structure, 224 binary images obtained by FIB/SEM were used. Figure 1c-i and Supplementary Movie 1a depict the projection images of the 3D reconstruction of the whole chromosome image. Figure 1c-ii and Supplementary Movie 1b also show the projection images of the 3D reconstruction of the chromosome virtually dissected by a tilted cutting plane. Careful observation of the 3D reconstructed chromosome revealed that it consisted of more or less evenly packed chromatin fibres in both chromatids and specific unevenly condensed or decondensed regions were not observed even at telomeric and centromeric regions. Under our experimental conditions, we did not observe any periodical pattern of chromatin arrangement.

**Figure 2:**
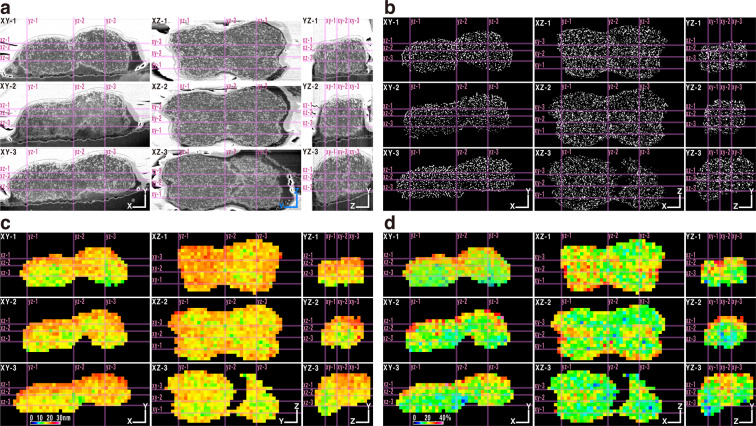
Chromosome cross-sections and heatmaps from three directions. (**a**), Cross-sectioned grey images from three different directions. (**b)**, Cross-sectioned binary images. (**c)**, Heatmaps of voxels (cubes 120-nm on each side) based on averaged diameters of chromatin fibres. (**d)**, Heatmaps of voxels based on averaged volume (%) of chromatin fibres by using ImageJ 1.51 with an in-house macro script. Bar = 500 nm.

To quantitatively confirm our findings, the overall distribution patterns of chromatin fibres were examined in greater detail in 3D. Figures 2a,b demonstrate the distributions of chromatin fibres within the chromosome interior using grey and binary images cut by three cutting planes for three directions. XZ and YZ images were reconstructed from a set of XY cross-section images. Supplementary Movies 2a and 2b present the stacked 224 XY cross-section images of grey and binary images, respectively. Supplementary Movie 2c demonstrates the location of thick chromatin fibres with more than 30-nm (coloured red). The thick fibres had a weak tendency to be distributed at the outer location of the chromosome; however, no periodical arrangements were observed. These data demonstrated random shape and random distribution of chromatin fibres in the chromosome interior and again no specific periodical pattern of chromatin distribution was observed in any of the three different directions. Furthermore, almost no thick chromatin fibre with more than 30-nm diameter could be observed. These tendencies were the same between the two sister chromatids and among chromosome interstitial regions, as well as telomeric and centromeric regions, although human chromosome 20 has a G-band positive region on both short and long arms^16^.

Next, we quantitatively measured the average diameter of chromatin fibres within cubes 120-nm on each side (1.73 × 10^6^ nm^3^). Figure 2c shows the heatmap of the distribution of the average diameter of chromatin fibres across the chromosome. When the chromatin diameter was approximately 10, 20, and 30-nm, the voxel was coloured light green, orange, and pink, respectively. The axial regions of both sister chromatids were mainly coloured light green (10-nm) to yellow (15-nm), as shown in Fig. 2c XZ-2. This was confirmed from the stack images of coloured voxels across the chromosome (Supplementary Movie 2c). The chromatin density (% volume) of each cube across the chromosome was examined in a heatmap (Fig. 2d). When chromatin density was approximately 10, 20, and 30%, the voxel was coloured cyan, yellow, and red, respectively. The axial regions of both sister chromatids were coloured green (15%), as shown in Fig. 2d XZ-2 and YZ-3. Supplementary Movie 2d demonstrates all voxels across the interior of the chromosome.

## Discussion

From our observations, the majority of the diameters of the inner chromatin fibre were found to be between 5–25-nm, whereas fibres more than 25-nm were in the minority. The majority presence of chromatin fibres less than 25-nm was consistent with recent results obtained by cryo-EM^6^ and ChromEM^12^, rejecting the hierarchical coiling model and its derivatives of nucleosomes-on-a-DNA strand forming to thicker chromatin structures. Previous studies have also shown that reconstructed nucleosomes indicate that the tetra-nucleosome unit has 27.2-nm in fibre diameter^18^, and Hi-CO analysis in yeast showed that the fibre-like nucleosome has peaks of 22–26-nm in thickness^19^. These evidences would support that the major chromatin diameters are less than 30-nm. In mitotic chromosomes, nucleosomes become more crowded than when in interphase, thereby allowing a nucleosome to interact with distant ones, thus decreasing both the interaction among neighbouring nucleosomes and the higher-order chromatin structures^20^. Consequently, chromatin fibres with a diameter larger than 30-nm were rarely observed (Fig. 1b), which is consistent with the loss of topologically associating domains (TADs) at the metaphase stage, as revealed by Hi-C analysis^10,21^.

In contrast to previous reports^7^, no periodical chromatin structures were observed in this study. An unstable order of fluorescent *in situ* hybridisation signals between chromatids of a metaphase chromosome with the probes closely located in the genome DNA^22^ would indicate random packing of chromatin fibres. However, it was expected that axially compressed chromatin loop arrays would be formed after loss of TADs, as in prophase nuclei, and that they would be arranged helically around the twisted chromosome scaffold^10,21^. Therefore, certain regularities of chromatin fibre might be observed in the chromosomes, especially around the chromosome scaffold. As the chromosome scaffold is known to be located around the central axis of each chromatid^20,23,24^, the lower values of the average diameter and the chromatin density would indicate that chromatin fibres were less condensed at the axial regions of both chromatids. These data clearly demonstrate the reduced density of chromatin at scaffold regions. It would be essential to examine whether this compartmentalisation based on chromatin density would simply occur by scrambling for space within the chromosome scaffold or through other structural reasons.

The inner structure of barley metaphase chromosome obtained by FIB/SEM showed that chromatin fibres occupied 81% of chromosome volume and cavities form a network throughout the chromosome arms^13^. In contrast to barley, our FIB/SEM observation of human metaphase chromosome showed that chromatin fibres occupied 19% of chromosome volume. This difference in chromatin density could be because plant chromosomes are densely condensed and contain high amounts of DNA per unit length compared with human chromosomes^25^. Estimations from DNA contents of each chromosome in human^17^ and barley^26^ and chromosome photographs of human^27–33^ and barley^34–37^ indicate that the DNA density per unit volume of barley metaphase chromosome would be up to approximately ten times as dense as that of human chromosomes. The FIB/SEM observations revealed that the difference in chromosome condensation between plant and human might be caused by the different arrangement of chromatin fibre in the inner space of the chromosome, suggesting a species-dependent inner structure of metaphase chromosome.

In conclusion, our results confirmed the presence of the 30-nm basic chromatin fibre; however, chromatin fibres with the diameter equal to or more than 30-nm would play a minor role in the construction of the chromosome inner structure, as they neither constituted the majority among chromatin fibres nor did they locate at specific regions. Instead, fibres with various diameters between 5–25-nm made up the majority of chromatin fibres of mitotic metaphase chromosome. Thus, our results reject the possibility for the use of the coiled-coil or hierarchical coiling models to explain the higher-order structure of chromosomes under the experimental conditions that we employed. Alternatively, the contribution of the minor presence of 30-nm fibres to the chromosome higher-order structure remains to be elucidated. Furthermore, the reduced density of chromatin fibres at axial regions clearly indicate that the overall distribution of chromatin fibres within a chromosome follow a certain folding pattern, which needs to be resolved through future research.

## Methods

### Preparation of chromosome samples

We employed the methods described in a previous manuscript^15^. Briefly, human metaphase chromosomes were isolated from HeLa S3 suspension cells using the polyamine method^38^, and stored in 70% glycerol at –20 °C. Chromosomes were dropped onto an aluminium substrate coated with 0.01% poly-l-lysine. After 10 min, the substrates were washed with XBE2 buffer (10 mM HEPES, 2 mM MgCl_2_, 100 mM KCl, and 5 mM EGTA, pH 7.7). The chromosomes were fixed with 2.5% glutaraldehyde for 30 min, washed 3 times with XBE2 buffer for 5 min and stained with Pt-blue (TI blue, Nisshin EM, No. 335) for 1 h. Although Pt-blue stains not only DNA but also RNA and two amino acids, affinity to DNA and RNA are much higher than the amino acids^39^. Ti blue has been shown to be effective for staining chromosomes^15,40^. After staining, the chromosomes were washed with XBE0 buffer (10 mM HEPES, 100 mM KCl, and 5 mM EGTA, pH 7.7) for 30 min. Then, 0.5% ionic liquid, 1-butyl-3-methylimidazolium tetrafluoroborate (BMIMBF_4_, C_8_H_15_BF_4_N_2_, Sigma-Aldrich, 91508) solution was dropped onto the chromosomes to keep chromosomes closer to the native state. Subsequently, the samples were subjected to semi-vacuum conditions for more than 1 h.

### FIB/SEM

Aluminium substrates carrying chromosomes were cut into a suitable size and fixed on an aluminium stub using electron conductive carbon tape^14,15^. After insertion of the stub with the chromosomes into the FIB/SEM system (Helios 660, FEI Inc.), platinum was deposited on target chromosomes. Chromosomes were then cut at every 10-nm interval by the gallium ion beam emitted from the perpendicularly positioned gun to the tilted stage. A total of 224 images of cross-sections were obtained using the secondary electron mode with an accelerating voltage of 2.0 keV.

### Imaging processes and analyses

For image analyses, ImageJ 1.51^41^ was used. The sample used for the analysis was the one which showed the typical pattern known from before^15^. A series of cross-section images obtained by SEM were employed for several imaging processes, such as conversion from 8-bit to 32-bit greyscale, equalisation of X and Y scales, manual selection of chromosomal areas, normalisation of grey values in chromosomal areas equalising values of mean and standard deviation to reduce unevenness between cross-sections, bandpass filtering (2–100 pixels) to reduce noise and unevenness within a cross-section, conversion from 32-bit to 8-bit grey, binarisation using a local thresholding method calculating the mean of a neighbourhood (radius = 11 pixels, c = –23), and manual refinement of the binary image set of chromatin fibres.

The axial line of each chromatin fibre was calculated from the binary image set by using plugins of ImageJ, the Chamfer Distance Map function in MorphoLibJ^42^ and the Ridge detection plugin^43^. The width of the chromatin fibre was measured as the length of perpendicular lines to the axial lines within each chromatin fibre using an in-house macro script. The number of measured points on the axial lines were 4,118,080.

Heatmaps of chromatin density and fibre width were drawn as 120 nm cubes in X, Y, and Z directions. Chromosomal area was determined from the binary image set of chromatin fibres by image processing using ImageJ. When more than 80% of total volume of the voxel was occupied by chromosomal volume, the voxel was attributed to chromosomal volume and was coloured in heatmaps according to its average chromatin diameter or chromatin density.

## Supplementary information

Supplementary Information.

Supplementary Information.

Supplementary Information.

Supplementary Information.

Supplementary Information.

Supplementary Information.

Supplementary Information.

Supplementary Information.

Supplementary Information.
