# Supplementary material for: Human metaphase chromosome consists of randomly arranged chromatin fibres with up to 30-nm diameter
Source: Sci Rep. 2020 Jun 2;10:8948. doi: 10.1038/s41598-020-65842-z (PMC7265543; doi:10.1038/s41598-020-65842-z)
Supplement: Supplementary file 1 — Supplementary Information. [file 41598_2020_65842_MOESM1_ESM.docx]

**Supplementary Information**

**Human metaphase chromosome consists of randomly arranged chromatin fibres with up to 30-nm diameter**

Toshiyuki Wako^1^, Akiyo Yoshida^2^, Jun Kato^2^, Yuji Otsuka^2^, Shinichi Ogawa^3^, Kohei Kaneyoshi^4^, Hideaki Takata^5^, and Kiichi Fukui^6*^

^1^ Institute of Crop Sciences, National Agriculture and Food Research Organization, 2-1-1 Kannondai, Tsukuba, Ibaraki 305-8602, Japan.

^2^ Morphological Research Laboratory, Toray Research Center Inc., 3-3-7 Sonoyama, Otsu, Shiga 520-8567, Japan.

^3^ Nanoelectronics Research Institute, National Institute of Advanced Industrial Science and Technology, 1-1-1 Umezono, Tsukuba, Ibaraki 305-8568, Japan.

^4^ Graduate School of Engineering, Osaka University, 2-1 Yamadaoka, Suita, Osaka 565-0871, Japan.

^5^ Kansai Center, National Institute of Advanced Industrial Science and Technology, Midorigaoka, Ikeda, Osaka 563-8577, Japan.

^6^ Graduate School of Pharmaceutical Sciences, Osaka University, 1-6 Yamadaoka, Suita, Osaka 565-0871, Japan.

*Corresponding author

Tel & Fax: +81-6-6879-7440

E-mail: [kfukui@bio.eng.osaka-u.ac.jp](mailto:kfukui@bio.eng.osaka-u.ac.jp)

ORCID: 0000-0002-9156-819X

**Figure legends for Supplementary Figure and Movies**

**Suppl. Fig. 1:** Scanning electron microscopy image of a human metaphase chromosome prepared on an aluminium substrate before focused ion beam (FIB) dissection. Bar = 1-µm.

**Suppl. Mov. 1: 3D reconstructed movies.** **a,** Movie of surface structure of the 3D reconstructed chromosome with various angles and directions. **b,** Movie enabling observation of the inner structure of the chromosome virtually dissected by tilted cutting planes.

**Suppl. Mov. 2:** **3D reconstructed movies of the cross-sections and heatmaps. a** and **b,** Movies for stacked grey and binary images from X-Y direction, respectively. **c**, Movie for stacked binary image of chromatin fibres with more than 30-nm diameter from X-Y direction (identified by red colour). **d** and **e,** Movies of heatmaps from X-Z direction depicting all stacked voxels (120-nm cubes), showing average chromatin diameters and average chromatin density (% volume) within individual voxels in different colours, respectively.


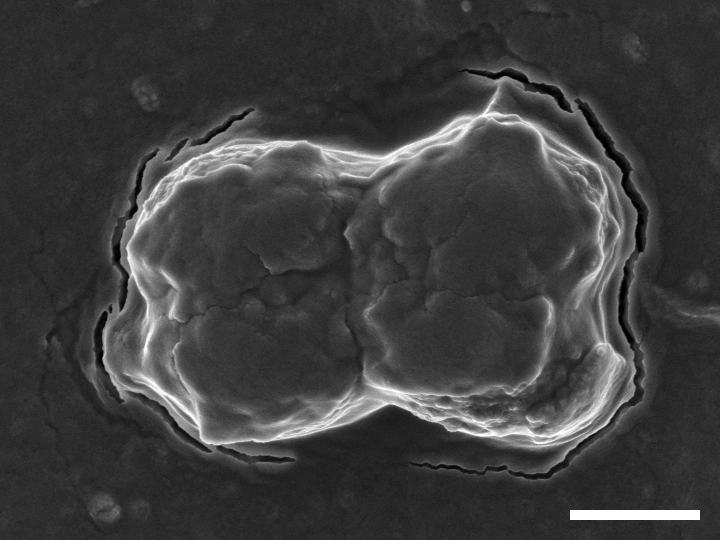


**Suppl. Fig. 1:** Scanning electron microscopy image of a human metaphase chromosome prepared on an aluminium substrate before focused ion beam (FIB) dissection. Bar = 1-µm.
