# Supplementary figures and images for: Human metaphase chromosome consists of randomly arranged chromatin fibres with up to 30-nm diameter
Source: Sci Rep. 2020 Jun 2;10:8948. doi: 10.1038/s41598-020-65842-z (PMC7265543; doi:10.1038/s41598-020-65842-z)

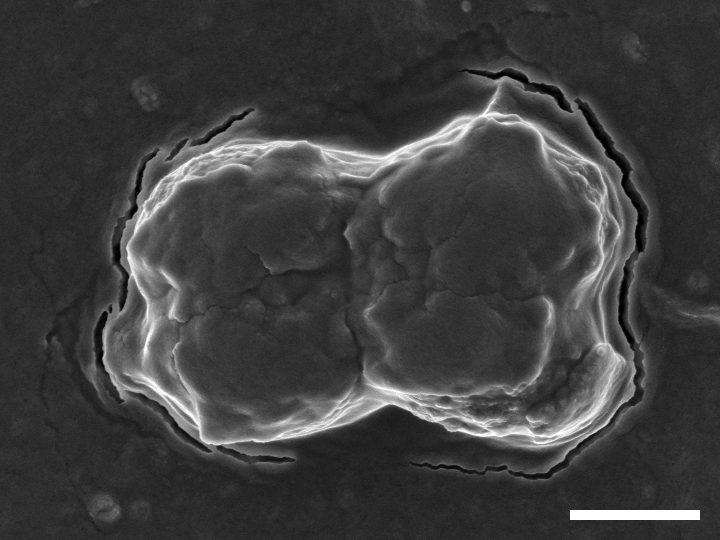

Supplement: Supplementary file 2 — Supplementary Information. [file 41598_2020_65842_MOESM2_ESM.png]
